# Coordinated Antioxidant and Physiological Responses at Flowering Promote Yield Stability in Salinity-Stressed Barley Genotypes

**DOI:** 10.3390/ijms27052454

**Published:** 2026-03-07

**Authors:** Faiza Boussora, Sihem Ben Ali, Tebra Triki, Amna Ghanmi, Mohamed Bagues, Ali Ferchichi, Ferdaous Guasmi

**Affiliations:** 1 Institute of Arid Lands of Medenine (IRA), Medenine 4119, Tunisia; 2 Higher Institute of Applied Biologyof Medenine (ISBAM), University of Gabes, Medenine 4119, Tunisia; 3 National Institute of Agronomic Research of Tunis (INAT), Tunis 1082, Tunisia

**Keywords:** salinity stress, barley, antioxidant defense, osmotic adjustment, phenolic compounds

## Abstract

Salinity stress severely limits barley production by disrupting physiological and biochemical processes critical for growth and yield. Although numerous studies have examined individual physiological or antioxidant responses to salinity, an integrated multivariate understanding of how these mechanisms collectively contribute to yield stability at the flowering stage remains limited. This study aimed to elucidate the integrated antioxidant and physiological mechanisms underlying salinity tolerance in barley genotypes during flowering. Barley plants were subjected to controlled salinity treatments, and a comprehensive set of phenolic compounds, antioxidant capacity indices, physiological traits, and yield components were measured. Multivariate analyses, including redundancy analysis (RDA) and partial least squares regression (PLSR), identified key traits contributing to yield stability under salinity. Multivariate analyses revealed also genotype-specific physiological strategies underlying contrasting salinity tolerance levels. Antioxidant defenses, such as total phenolics, DPPH and ABTS radical scavenging activities, and α-tocopherol, along with osmotic regulators like proline and soluble sugars, were closely associated with improved water status and reduced oxidative damage. These coordinated responses correlated strongly with yield components, including thousand-grain weight and main spike seed number. Notably, this study provides new insights into the predictive relevance of selected biochemical and physiological markers for yield performance under salt stress using PLSR at the flowering stage. PLSR further demonstrated the high predictive power of a limited subset of biochemical and physiological markers for yield traits under salt stress. Collectively, these findings reveal that the interplay between antioxidant machinery and osmotic adjustment at flowering is critical for barley resilience to salinity, providing valuable physiological markers to inform breeding strategies aimed at improving salt tolerance.

## 1. Introduction

Soil salinity is one of the most severe abiotic constraints limiting agricultural productivity worldwide, affecting more than 20% of irrigated lands and posing a major threat to food security [1]. This problem is particularly acute in arid and semi-arid regions, where low rainfall, high evapotranspiration rates, saline groundwater, and inappropriate irrigation practices accelerate soil salinization [2]. In North Africa, and especially in southern Tunisia, salinity represents a chronic and expanding constraint that severely restricts cereal production and threatens the sustainability of rainfed and irrigated farming systems [3,4]. Southern Tunisia is characterized by an arid climate, scarce and irregular precipitation, high temperatures, and widespread use of saline water for irrigation [5,6]. Under these conditions, the accumulation of salts in agricultural soils has become unavoidable, leading to reduced crop establishment, impaired plant growth, and unstable yields [7,8]. Developing crop varieties capable of maintaining productivity under saline conditions is therefore a strategic priority for ensuring food security and sustaining rural livelihoods in this region.

Among cereal crops, barley (*Hordeum vulgare* L.) occupies a central position in the agricultural systems of arid and marginal environments [9]. In southern Tunisia, barley is widely cultivated due to its relatively low input requirements, adaptability to poor soils, and importance as a staple food and animal feed [10]. Beyond its agronomic value, barley grain is nutritionally rich, providing carbohydrates, proteins, dietary fibers, minerals, and bioactive compounds with recognized health benefits [11,12]. Its moderate tolerance to salinity, compared to other cereals, makes barley a key candidate for cultivation in salt-affected areas and a valuable model species for studying stress tolerance mechanisms [13,14].

Salinity stress imposes a complex combination of osmotic and ionic effects on plants [15]. The initial osmotic phase restricts water uptake and reduces cellular turgor [16,17], while the subsequent ionic phase leads to excessive accumulation of toxic ions, particularly Na^+^ and Cl^−^, causing membrane destabilization, enzyme inhibition, and nutrient imbalance [18]. These constraints ultimately converge in oxidative stress, characterized by the overproduction of reactive oxygen species (ROS) such as superoxide radicals, hydrogen peroxide, and hydroxyl radicals [19]. If not efficiently controlled, ROS cause lipid peroxidation, protein degradation, and nucleic acid damage, resulting in cellular dysfunction and yield reduction [19,20].

To cope with salt-induced oxidative stress, plants activate an intricate antioxidant defense system composed of enzymatic and non-enzymatic components. While antioxidant enzymes constitute the first line of defense, secondary metabolites, especially phenolic compounds, play a pivotal role in maintaining redox balance [21]. Phenolic acids, flavonoids, and tannins contribute to stress tolerance by scavenging ROS, strengthening cell walls, chelating metal ions, and modulating stress-responsive signaling pathways [22,23].

In barley, phenolic acids such as quinic, protocatechuic, syringic, p-coumaric, and trans-ferulic acids represent major constituents of the phenolic pool and have been associated with antioxidant capacity and stress adaptation [24,25]. Hence, in response, plants enhance the biosynthesis of antioxidant metabolites that contribute to redox homeostasis and cellular protection. Phenolic compounds, including phenolic acids, flavonoids, and tannins, represent a major component of this defense system [22,25]. Their accumulation under salt stress enhances the plant’s capacity to neutralize free radicals and buffer oxidative pressure, as reflected by increased radical-scavenging activity measured through DPPH and ABTS assays [26]. This elevated scavenging capacity limits oxidative damage to membranes, proteins, and nucleic acids [27,28]. Beyond their antioxidant function, phenolic compounds also contribute to cell wall strengthening and reduced ion leakage, and they act as signaling molecules that modulate stress-responsive gene expression and metabolic adjustments during salinity acclimation [29,30].

In parallel with metabolic adjustments, physiological responses are crucial for maintaining plant function under salt stress. Osmotic adjustment through the accumulation of compatible solutes, such as proline and soluble sugars, helps preserve cellular hydration, stabilize proteins and membranes, and mitigate osmotic imbalance. Relative water content (RWC) serves as an integrative indicator of plant water status, whereas malondialdehyde (MDA) accumulation reflects the extent of membrane lipid peroxidation and oxidative damage [7,25,31].

Despite extensive research on salinity tolerance in barley, most studies have focused on early developmental stages, particularly germination and seedling growth. However, in arid regions such as southern Tunisia, yield instability is often determined by stress events occurring at the reproductive phase. The flowering stage is especially vulnerable, as it encompasses pollen development, fertilization, and grain set. Stress exposure during this phase can result in spikelet abortion, reduced grain number, impaired grain filling, and substantial yield losses [32,33].

Recent evidence suggests that stress responses at flowering involve distinct metabolic and physiological adjustments that cannot be fully inferred from vegetative-stage studies [34]. In this context, antioxidant capacity and secondary metabolite accumulation at flowering may play a decisive role in protecting reproductive tissues and sustaining yield formation under salinity. However, the relationships between antioxidant machinery, physiological stress indicators, and yield components at this critical stage remain poorly understood, particularly under the environmental conditions of arid and saline regions.

Therefore, the present study aimed to elucidate the biochemical and physiological mechanisms underlying salt resilience in five barley genotypes cultivated under conditions relevant to southern Tunisia, with a specific focus on the flowering stage. We quantified key phenolic acids, total polyphenols, flavonoids, and tannins, and evaluated antioxidant activity using DPPH and ABTS assays, along with α-tocopherol content. Physiological stress indicators, including relative water content, proline, soluble sugars, and malondialdehyde, were assessed in parallel. Yield-related traits, flowering date (FD), main spike number of seeds (MSGN), thousand grain weight (TGW), and main spike grain weight (MSGW), were measured to evaluate reproductive performance and yield stability under salinity.

## 2. Results

### 2.1. Total Phenolic and Flavonoid Contents

**Figure 1:**
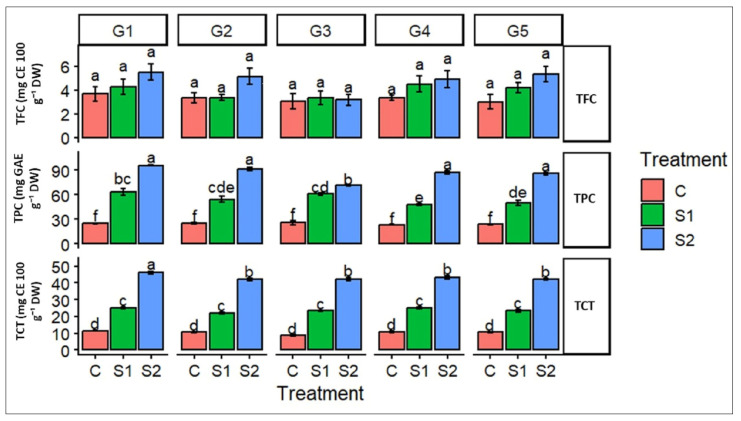
Effects of salinity stress on phenolic compound accumulation in barley genotypes. The figure illustrates the variation in total polyphenols (TPC), total flavonoids (TFC), and total tannins (TCT) measured in five barley genotypes: G1 (Ardhaoui), G2 (Kounouz), G3 (Lemsi), G4 (Manel), and G5 (Rihane) grown under three salinity treatments: control (C), moderate salinity (S1, 6 g L^−1^ NaCl), and high salinity (S2, 12 g L^−1^ NaCl). Values represent means ± standard error (SE). Different letters above the bars indicate significant differences among genotype × treatment combinations according to Tukey’s post hoc test at *p* < 0.05.

To elucidate the biochemical adjustments associated with salinity tolerance, the accumulation of major phenolic compounds was evaluated across barley genotypes subjected to increasing salinity levels, as shown in Figure 1.

Salinity stress distinctly influenced the accumulation of phenolic compounds across the studied barley genotypes. While total flavonoid content (TFC) showed only a slight, non-significant increase under moderate and severe salinity, indicating a relatively stable or constitutive regulation, both total polyphenol content (TPC) and total tannin content (TCT) responded strongly to salt stress. In all genotypes, plants exposed to high salinity (S2) showed significantly higher TPC and TCT levels compared to control conditions, with moderate salinity (S1) inducing intermediate responses. Among the measured traits, tannins displayed the most pronounced sensitivity to salinity, suggesting their pivotal role in stress-induced biochemical adjustment. The consistent enhancement of polyphenols and tannins under saline conditions highlights their contribution to the antioxidant defense system, likely involved in mitigating oxidative damage associated with salt stress, while the magnitude of the response varied moderately among genotypes.

### 2.2. Phenolic Compounds Variation

Salinity stress induced pronounced and genotype-dependent variations in individual phenolic acids detected by HPLC–MS analysis (Table 1).

Salinity stress induced pronounced and genotype-dependent variations in individual phenolic acids detected by HPLC–MS analysis. Quinic acid was the most abundant compound across all genotypes and treatments, although its accumulation showed contrasting responses to increasing salinity. While quinic acid content decreased under severe salinity (S2) in genotypes G1, G3, and G4, it markedly increased in G2 and G5, indicating differential metabolic regulation among genotypes. Protocatechuic acid generally increased under high salinity in G2, G3, and G4, whereas a decline was observed in G1 and G5, reflecting genotype-specific sensitivity to salt stress. Syringic acid exhibited a strong induction under S2 in most genotypes, particularly in G2 and G3, suggesting its involvement in stress-responsive phenylpropanoid pathways. In contrast, trans-ferulic acid showed a substantial accumulation under severe salinity in G1 and G2, while a moderate reduction or stabilization was observed in G3, G4, and G5. Similarly, p-coumaric acid displayed marked variability, with pronounced increases under S2 in G2 and G3, whereas reductions were detected in G1 and G5. Overall, the contrasting accumulation patterns of phenolic acids under increasing salinity highlight a complex, genotype-dependent reprogramming of secondary metabolism, likely contributing to differential stress adaptation mechanisms.

### 2.3. Antioxidant Activities

**Figure 2:**
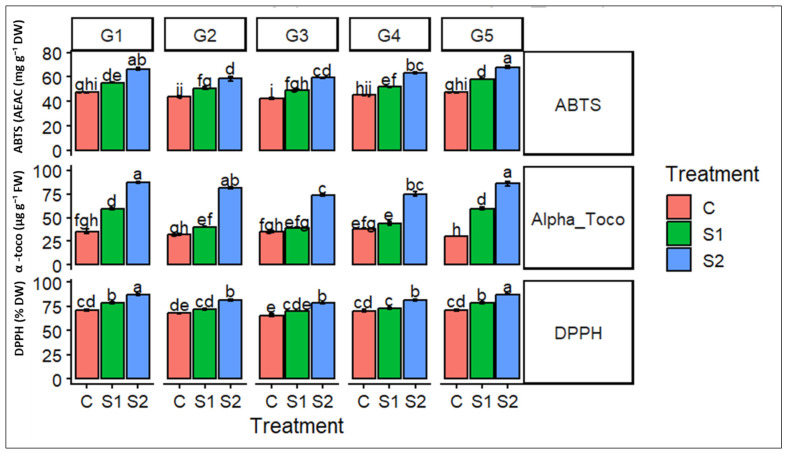
Effects of salinity stress on antioxidant capacity in barley genotypes. The figure presents ABTS radical scavenging activity, DPPH free radical scavenging activity, and α-tocopherol (Alpha-Toco) content measured in five barley genotypes: G1 (Ardhaoui), G2 (Kounouz), G3 (Lemsi), G4 (Manel), and G5 (Rihane) grown under three salinity treatments: control (C), moderate salinity (S1, 6 g L^−1^ NaCl), and high salinity (S2, 12 g L^−1^ NaCl). Bars represent mean values ± standard error (SE). Different letters above the bars indicate significant differences among genotype × treatment combinations according to Tukey’s post hoc test at *p* < 0.05.

The antioxidant capacity of barley genotypes under salinity stress was assessed through complementary non-enzymatic indicators, including ABTS radical scavenging activity, DPPH free radical scavenging capacity, and α-tocopherol (Alpha-Toco) content (Figure 2).

Across all genotypes, salinity stress significantly enhanced antioxidant activity, with the highest values consistently recorded under severe salinity (S2). ABTS activity showed a progressive increase from control to S2, with pronounced genotype-dependent differences, indicating differential efficiency in neutralizing hydrophilic radicals. Similarly, DPPH scavenging capacity increased significantly under salinity stress, particularly under S2, reflecting an enhanced ability to counteract stable free radicals. In parallel, α-tocopherol content exhibited a strong induction in stressed plants, especially in genotypes G1, G4, and G5, suggesting an important role of this lipophilic antioxidant in membrane protection under salt stress. Overall, the coordinated upregulation of radical scavenging activities and α-tocopherol accumulation under salinity highlights the activation of a robust antioxidant defense system, with marked variability among genotypes, potentially contributing to differential salinity tolerance.

### 2.4. Stress-Related Metabolites

**Figure 3:**
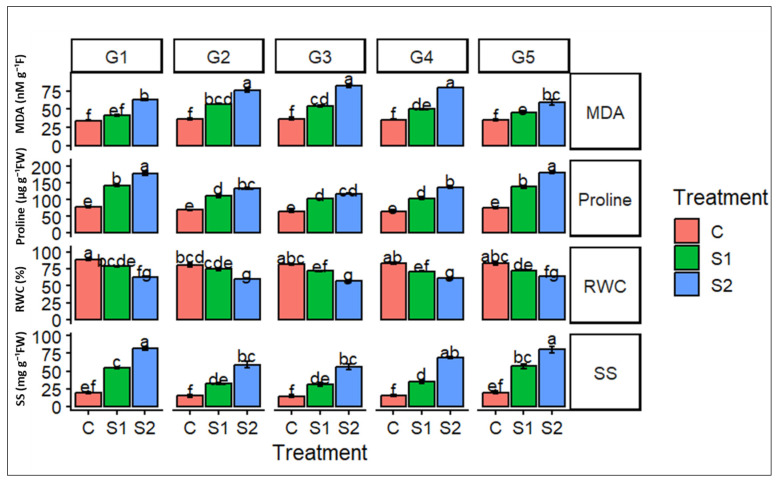
Effects of salinity stress on stress-related metabolites in barley genotypes. Measured traits include malondialdehyde (MDA), proline content, relative water content (RWC), and soluble sugars (SS). Five barley genotypes—G1 (Ardhaoui), G2 (Kounouz), G3 (Lemsi), G4 (Manel), and G5 (Rihane)—were grown under three salinity treatments—control (C), moderate salinity (S1, 6 g L^−1^ NaCl), and high salinity (S2, 12 g L^−1^ NaCl). Bars represent mean values ± standard error (SE). Different letters above the bars indicate significant differences among genotype × treatment combinations according to Tukey’s post hoc test at *p* < 0.05.

Salinity stress induced marked physiological responses across the evaluated barley genotypes (Figure 3). Increasing salinity from 6 g L^−1^ (S1) to 12 g L^−1^ (S2) resulted in a progressive and genotype-dependent increase in malondialdehyde (MDA) and proline contents, indicating intensified oxidative stress and the activation of osmotic adjustment mechanisms under saline conditions. The highest values were generally recorded at S2, with Lemsi (G3) and Rihane (G5) representing particularly elevated levels, suggesting a stronger stress perception and cellular perturbation in these genotypes. Conversely, relative water content (RWC) decreased with increasing salinity, reflecting impaired tissue hydration and altered water status, with the most pronounced reductions observed under high salinity (S2). In parallel, soluble sugars (SS) accumulated significantly in response to salinity stress, especially at S2, highlighting their potential role in osmoprotection, metabolic adjustment, and maintenance of cellular homeostasis. Overall, significant differences among genotypes, as indicated by the statistical grouping letters, emphasize the existence of diverse physiological strategies underlying salinity tolerance in barley.

### 2.5. Yield Components

**Figure 4:**
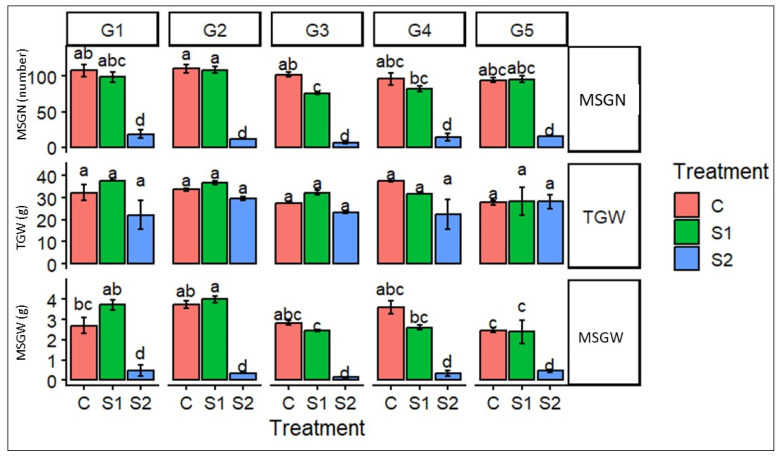
Effects of salinity stress on yield-related traits of barley genotypes. Main spike grain number (MSGN), thousand grain weight (TGW(g)), and main spike grain weight (MSGW) of five barley genotypes (G1–G5) grown under control (C), moderate salinity (S1), and severe salinity (S2) conditions. Bars represent mean values ± standard error. Different lowercase letters indicate statistically significant differences among treatments within each genotype (*p* < 0.05). Salinity stress, particularly under S2, markedly reduced reproductive and yield traits, with pronounced genotype-dependent responses.

The impact of salinity stress on yield-related and phenological traits of barley genotypes was evaluated through main spike seed number (MSGN), thousand grain weight (TGW), and main spike grain weight (MSGW) (Figure 4).

Across all genotypes, salinity stress markedly affected yield performance, with a general decline observed under severe salinity (S2). MSGN showed a pronounced reduction under S2 in all genotypes, indicating strong sensitivity of reproductive development to high salinity levels, while moderate salinity (S1) induced genotype-dependent responses, with some genotypes maintaining values comparable to the control. TGW exhibited comparatively greater stability under salinity stress, with only moderate reductions under S2 and minimal differences between control and S1 treatments, suggesting partial tolerance of grain filling processes to salt stress. In contrast, grain weight was strongly reduced under severe salinity, reflecting the cumulative negative effects of stress on assimilate partitioning and seed development. Notably, significant genotype-dependent variation was observed for all traits, with genotypes G1, G4, and G5 showing relatively better performance under stress conditions. Overall, the differential responses of reproductive and yield components under salinity stress highlight distinct strategies of stress tolerance among barley genotypes, likely associated with their physiological and biochemical adaptive capacities.

### 2.6. Multivariate Analysis

#### 2.6.1. Correlations

**Figure 5:**
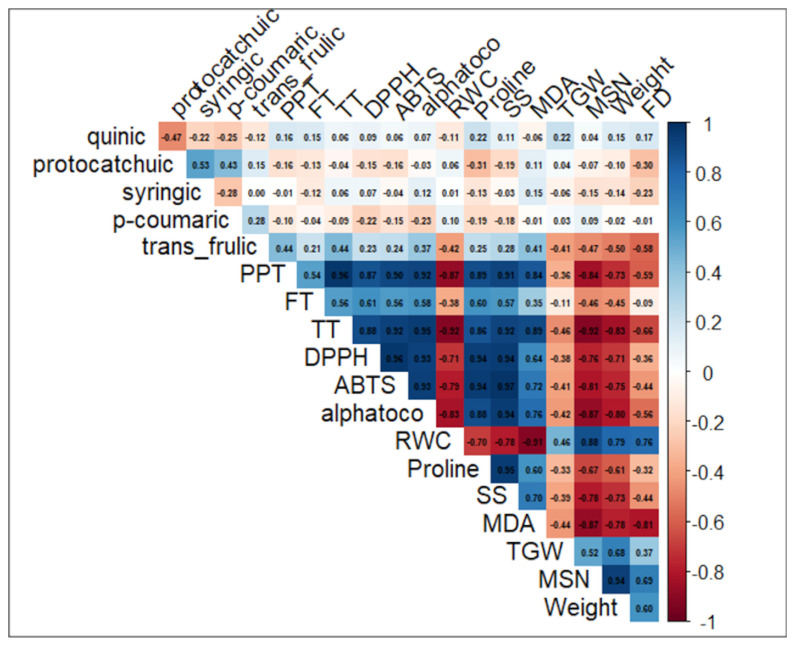
Total correlation matrix of antioxidant, physiological, and agronomic traits in salinity-stressed barley. Total Pearson correlation matrix showing relationships among individual phenolic compounds (quinic, protocatechuic, syringic, *p*-coumaric, and trans-ferulic acids), antioxidant-related parameters (PPT, FT, TT, DPPH, ABTS, and α-tocopherol), physiological traits (relative water content (RWC), proline, soluble sugars (SS), and malondialdehyde (MDA)), and agronomic traits (thousand grain weight (TGW), main spike seed number (MSGN), MSGW, and flowering duration (FD)) in barley subjected to salinity stress. Blue and red colors indicate positive and negative correlations, respectively, with color intensity proportional to correlation strength. The matrix highlights coordinated antioxidant responses and their associations with yield-related traits under saline conditions.

Figure 5 illustrates the total correlation matrix among individual phenolic compounds (quinic, protocatechuic, syringic, p-coumaric, and trans-ferulic acids), antioxidant-related metrics (PPT, FT, TT, DPPH, ABTS, and α-tocopherol), as well as physiological and agronomic traits (RWC, proline, soluble sugars (SS), MDA, TGW, MSGN, MSGW, and FD) in barley subjected to salinity stress.

Strong positive correlations were observed between total phenolic-related traits and antioxidant capacity indicators (PPT, FT, TT, DPPH, and ABTS), highlighting a tight coupling between phenolic accumulation and the activation of antioxidant defenses under saline conditions. Likewise, key yield components such as TGW, MSGN, and MSGW showed significant positive intercorrelations, suggesting a coordinated regulation of yield formation (Figure 5).

In contrast, pronounced negative correlations were detected between oxidative stress markers, particularly MDA, and several physiological and agronomic traits. MDA showed strong negative associations with RWC, TGW, and grain weight, indicating that increased lipid peroxidation is closely linked to impaired water status and yield reduction. Proline and soluble sugars were generally positively correlated with antioxidant-related traits, supporting their role as osmo-protectants and stress-adaptive metabolites (Figure 5). Overall, the correlation matrix reveals the existence of strong functional interactions among secondary metabolites, antioxidant responses, and agronomic performance in barley under salinity stress.

**Figure 6:**
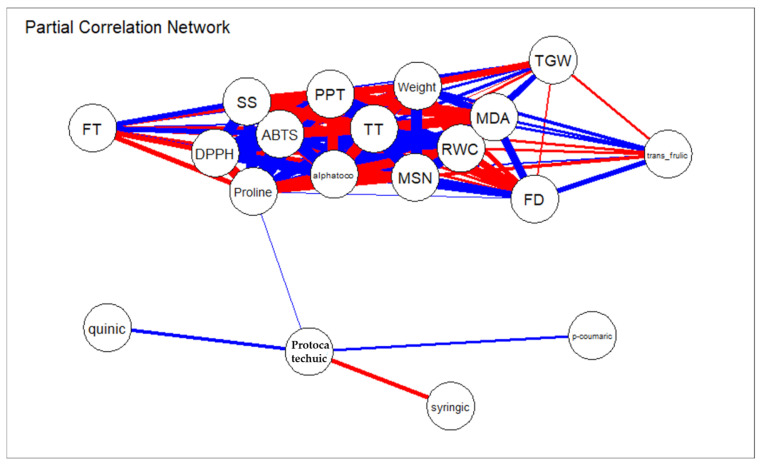
Partial correlation network revealing direct trait interactions under salinity stress. Partial correlation network illustrating direct associations among phenolic compounds, antioxidant capacity indices, physiological parameters, and agronomic traits in salinity-stressed barley after removing indirect effects. Nodes represent individual traits, while edges indicate significant partial correlations, with blue and red lines denoting positive and negative relationships, respectively. The network reveals a highly interconnected core linking antioxidant activity, osmotic adjustment, water status, and yield components, while oxidative stress (MDA) displays negative associations with performance-related traits.

Furthermore, the partial correlation network was constructed from the same set of traits, highlighting only direct relationships after removing indirect effects mediated by other traits (Figure 6).

A densely connected core network emerged, encompassing PPT, FT, TT, DPPH, ABTS, α-tocopherol, proline, soluble sugars, RWC, MSGN, TGW, and MSGW. This highly integrated cluster reflects a functional network linking antioxidant activity, osmotic adjustment, water status maintenance, and yield-related traits. Notably, the central position of RWC within this network underscores the pivotal role of plant water status in coordinating stress tolerance mechanisms and sustaining productivity under salinity (Figure 6).

Conversely, individual phenolic acids such as protocatechuic, syringic, quinic, and *p*-coumaric acids formed a more peripheral sub-network, showing relatively few direct connections with agronomic traits. This pattern suggests that their influence on plant performance is likely mediated through total phenolic content or global antioxidant activity rather than through direct effects on yield components. MDA emerged as a key negatively connected node, reinforcing its role as a central indicator of oxidative damage and its antagonistic relationship with physiological stability and yield performance (Figure 6).

Taken together, the combined analysis of total and partial correlations demonstrates that barley tolerance to salinity stress relies on a dynamic balance between phenolic accumulation, activation of antioxidant systems, maintenance of plant water status, and osmotic adjustment through proline and soluble sugars. Genotypes capable of simultaneously strengthening these interconnected mechanisms are better able to preserve yield components (MSGN, TGW, and MSGW) while limiting lipid peroxidation, as reflected by reduced MDA levels.

Genotype-wise correlation analyses revealed marked differences in trait–yield relationships (Figures S1 and S2). Tolerant genotypes showed strong positive correlations between yield components and antioxidant-related traits, whereas sensitive genotypes showed weaker or inconsistent associations, often coupled with elevated oxidative stress indicators. In G1 (Ardhaoui) and G2 (Kounouz), yield components (FD, MSGN, TGW and MSGW) were strongly and negatively correlated with oxidative stress markers (MDA), osmolytes (proline and soluble sugars), and antioxidant activities (DPPH, ABTS and α-tocopherol), while showing consistent positive correlations with relative water content (RWC). In contrast, G3 (Lemsi) displayed a more heterogeneous correlation pattern, with some phenolic compounds (e.g., quinic and p-coumaric acids) showing positive associations with TGW, whereas oxidative stress-related traits remained negatively correlated with most yield components.

G4 (Manel) displayed a distinct profile characterized by strong positive correlations between RWC and all yield components, while correlations with stress-related biochemical traits were generally weaker, indicating greater yield stability. In G5 (Rihane), specific phenolic compounds (notably syringic acid) were positively correlated with certain yield components, whereas MDA and proline maintained negative associations with yield.

#### 2.6.2. Principal Components Analysis PCA

**Figure 7:**
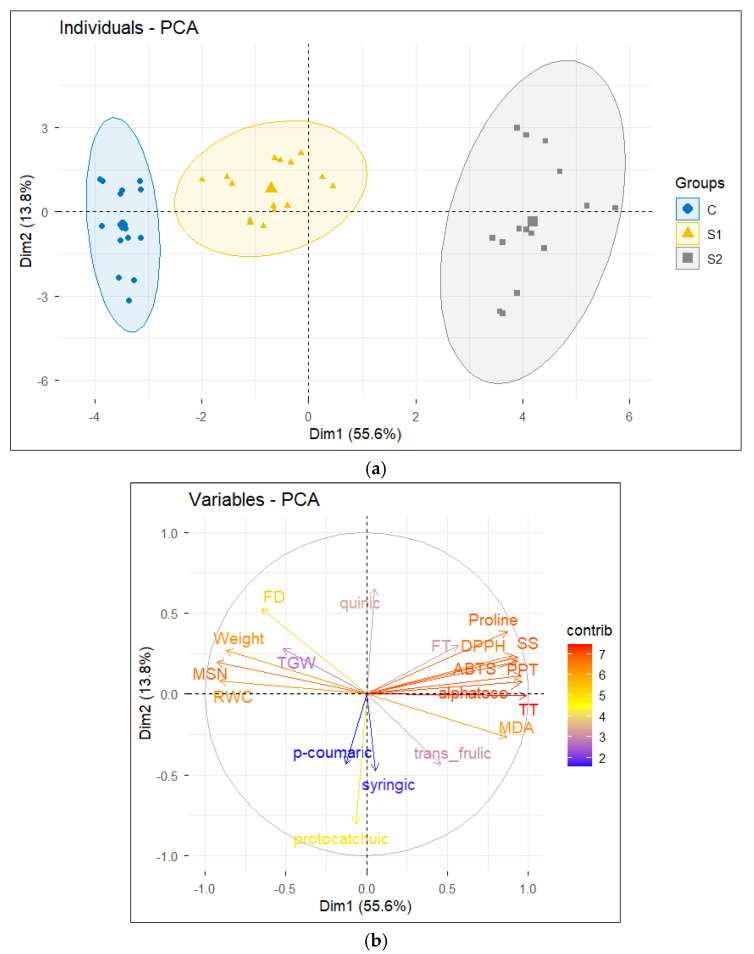
Principal component analysis (PCA) of individuals and traits in salinity-stressed barley. Principal component analysis (PCA) performed on phenolic compounds, antioxidant capacity indices, physiological parameters, and agronomic traits in barley exposed to salinity stress. (**a**) Projection of individuals (genotypes × treatments) onto the first two principal components, illustrating treatment- and genotype-dependent responses to salinity. (**b**) Projection of variables, showing the contribution and relationships of antioxidant defenses, osmoprotective traits, water status, and yield-related parameters. The first two principal components summarize the major sources of variation and highlight the trade-off between stress defense mechanisms and agronomic performance under increasing salinity.

The projection of individuals onto the factorial plane defined by the first two principal components (Dim1 = 55.6%; Dim2 = 13.8%) revealed a clear and well-structured separation among salinity treatments, indicating a strong effect of salt stress on barley genotypes. Control plants (C) clustered predominantly on the negative side of Dim1, reflecting a favorable physiological status characterized by higher water content and better growth and yield performance. Individuals subjected to moderate salinity stress (S1, 6 g L^−1^) occupied an intermediate position along Dim1, suggesting a partial adaptive response to salinity. In contrast, plants exposed to severe salinity stress (S2, 12 g L^−1^) were strongly projected on the positive side of Dim1, indicating pronounced physiological and metabolic alterations under high salinity conditions (Figure 7a).

Dim2 mainly captured intra-treatment variability, likely reflecting genotype-dependent differences in salinity tolerance. Within the S2 treatment, certain genotypes exhibited positive scores along Dim2, suggesting a more efficient activation of stress defense mechanisms, whereas others showed greater sensitivity. The increased dispersion observed under severe stress highlights the existence of contrasting physiological and biochemical strategies among barley genotypes when exposed to identical saline conditions, revealing a substantial potential for selecting salt-tolerant genotypes (Figure 7a).

The PCA of variables revealed two antagonistic trait groups along Dim1. On the positive side of this axis, oxidative stress markers and adaptive response traits—including MDA, proline, soluble sugars (SS), DPPH, ABTS, PPT, FT, TT, and α-tocopherol—were strongly and positively associated. Their orientation toward S2 individuals indicates an enhanced accumulation of antioxidant and osmoprotective compounds, reflecting an intensified biochemical response to salinity stress. Phenolic acids such as trans-ferulic, syringic, and p-coumaric acids also contributed to this defensive axis, supporting their role in mitigating oxidative damage under saline conditions (Figure 7b).

Conversely, agronomic and water-related traits (RWC, TGW, MSGN, MSGW, and flowering duration) were projected on the negative side of Dim1 and were primarily associated with control plants (Figure 7b). This pattern indicates that increasing salinity leads to a reduction in plant water status, growth, and yield components. Dim2 was more strongly influenced by specific phenolic acids, particularly quinic and protocatechuic acids, suggesting finer-scale metabolic adjustments that are likely genotype-dependent. Overall, the PCA highlights a clear trade-off between agronomic performance and the activation of stress defense mechanisms under salinity.

#### 2.6.3. Redundancy Analysis RDA

**Figure 8:**
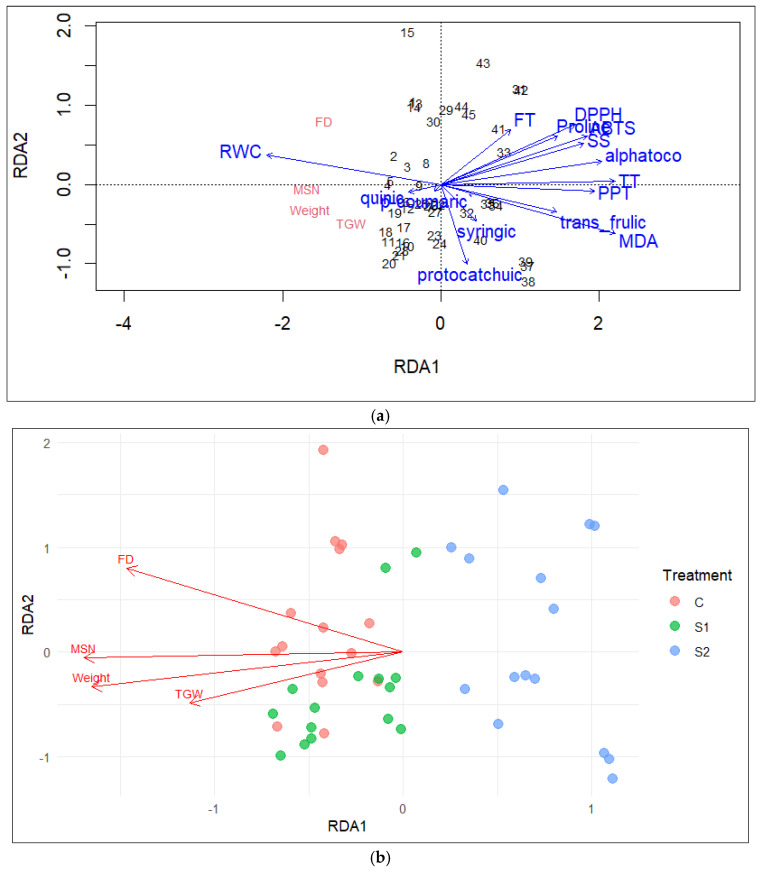
Redundancy analysis (RDA) of antioxidant, physiological, and yield traits in barley under salinity stress. (**a**) RDA ordination integrating antioxidant compounds (phenolic acids, DPPH, ABTS, and α-tocopherol), physiological traits (relative water content, proline, soluble sugars, and malondialdehyde), and yield-related variables (thousand grain weight, main spike seed number, grain weight, and flowering date) in barley subjected to salinity stress. Only traits showing significant contributions to the RDA model were retained. Arrows indicate the direction and strength of correlations with the ordination axes, and points represent individual observations. (**b**) Simplified RDA ordination highlighting treatment-driven separation of barley genotypes under control (C), moderate salinity (S1), and severe salinity (S2) conditions, based primarily on yield-related traits. This combined representation illustrates both the mechanistic relationships among stress-responsive traits and the agronomic consequences of increasing salinity stress.

Redundancy analysis (RDA) was conducted to unravel the multivariate relationships between yield components, physiological traits, and antioxidant responses of barley under salinity stress. The first two constrained axes explained a significant proportion of the variance, underscoring the strong influence of salinity on trait coordination and genotype differentiation. The RDA model was tested for significance using permutation-based ANOVA. The first canonical axis was highly significant (F = 392.52, *p* = 0.001), explaining the majority of constrained variation, whereas the second axis was not significant (*p* = 0.503). This confirms the strong relationship between the explanatory variables and the response traits primarily captured by the first axis. Key antioxidant traits—including phenolic acids, radical scavenging activities (DPPH, ABTS), and α-tocopherol—alongside oxidative stress marker MDA and water-status indicators such as relative water content (RWC), proline, and soluble sugars, were strongly associated with the main RDA axes. This suggests their pivotal role in the biochemical and physiological adjustments to salinity stress and their linkage to yield stability (Figure 8a).

Yield-related traits (thousand-grain weight, mean spike number, grain weight) were negatively correlated with the salinity gradient and clustered with control conditions, reflecting the adverse impact of salt stress on productivity. A secondary, simplified ordination highlighted the clear separation of treatments based predominantly on yield components, facilitating interpretation of agronomic responses but omitting the underlying physiological complexity. Together, these analyses reveal that barley salinity tolerance and yield resilience rely on a coordinated network of antioxidant and physiological traits, with the full RDA elucidating mechanistic pathways and the simplified ordination illustrating agronomic outcomes (Figure 8b).

#### 2.6.4. Partial Least Squares Regression (PLSR)

**Figure 9:**
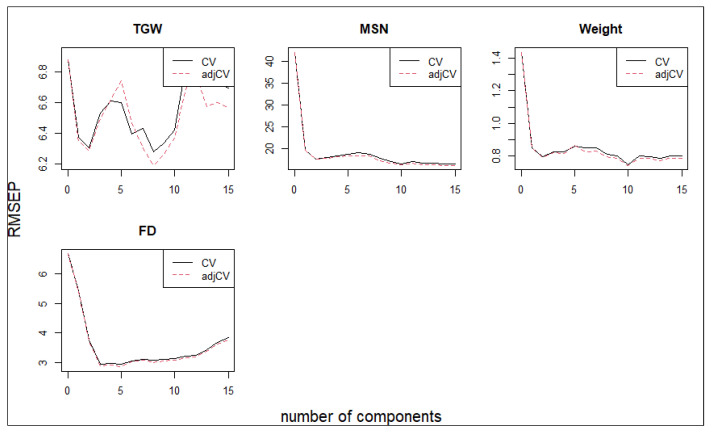
Cross-validated partial least squares regression (PLSR) models predicting barley yield traits from antioxidant and physiological variables under salinity stress. Cross-validation plots showing the root mean square error of prediction (RMSEP) as a function of the number of latent components in partial least squares regression (PLSR) models for yield-related traits, including thousand grain weight (TGW), main spike seed number (MSGN), MSGW, and flowering date (FD). Solid lines represent cross-validated RMSEP values, while dashed lines indicate bias-corrected RMSEP (adjCV). The rapid decrease and subsequent stabilization of RMSEP values indicate that a limited number of latent components is sufficient to achieve robust prediction, demonstrating the strong predictive capacity of antioxidant and physiological traits for yield performance under salinity stress.

Partial least squares regression (PLSR) was applied to evaluate the predictive capacity of antioxidant and physiological traits for yield-related components in barley subjected to salinity stress (Figure 9).

For each yield trait, the optimal number of latent variables was selected based on the lowest Root Mean Square Error of Prediction (RMSEP). For Thousand Grain Weight (TGW), the optimal number of components was 11, yielding an R^2^ of 0.574 and an RMSEP of 20.995. Similar statistics were obtained for other traits (e.g., MSGN: 13 components, R^2^ = 0.935, RMSEP = 121.922; FD: 9 components, R^2^ = 0.866, RMSEP = 6.051; MSGW: 11 components, R^2^ = 0.876, RMSEP = 0.267). Cross-validation analysis revealed a marked reduction in RMSEP with the inclusion of the first latent components, followed by stabilization or slight increases thereafter, indicating an optimal and limited number of components for reliable prediction. Thousand grain weight (TGW), main spike seeds number (MSGN), MSGW, and flowering date (FD) were accurately predicted using a small set of latent variables, suggesting that yield variation under salinity stress is primarily driven by a restricted subset of stress-responsive traits (Figure 9). The close agreement between cross-validated and bias-corrected RMSEP values further supports the robustness of the PLSR models. Variable Importance in Projection (VIP) scores identified key biochemical and physiological predictors contributing most to the models, highlighting, for instance, [insert top predictors]. The VIP plots (Figure S3) illustrate these influential variables for each yield component. These results demonstrate that antioxidant capacity indices, phenolic compounds, and physiological stress markers collectively provide strong predictive power for yield performance under saline conditions, complementing the mechanistic relationships revealed by redundancy analysis.

## 3. Discussion

Salinity stress represents a major abiotic constraint limiting barley productivity by imposing osmotic, ionic, and oxidative challenges that disrupt plant physiological and metabolic homeostasis. Our comprehensive multivariate analyses elucidate how barley genotypes orchestrate integrated responses involving antioxidant defenses, osmotic regulation, and water status maintenance to mitigate the deleterious effects of salinity and preserve yield stability.

The osmotic component of salt stress reduces water availability, leading to decreased relative water content (RWC) and cellular dehydration [7,35]. Under salinity stress, the reduction in leaf water potential and osmotic potential compels plants to adjust osmotically to maintain turgor pressure and cell volume, thereby preserving physiological processes [36,37]. This osmotic adjustment involves the accumulation of compatible solutes such as proline and soluble sugars, which lower the cellular osmotic potential, enabling continued water uptake despite the increasingly negative water potential in the rooting medium [38,39]. Maintenance of water status, as reflected by relative water content (RWC), emerged as a central pivot within the physiological network, corroborated by its key position in both the partial correlation network and redundancy analysis [25,40]. The coordinated accumulation of osmolytes, including proline and soluble sugars, alongside effective antioxidant responses, plays a pivotal role in maintaining cellular hydration and metabolic activity under salinity stress, which is critical for successful grain filling and biomass production [41]. Recent research has highlighted proline as a key compatible solute that not only contributes to osmotic adjustment by maintaining cell turgor but also acts as a potent antioxidant, stabilizing proteins and membranes under stress conditions [15,42]. Our findings align with these studies, demonstrating a significant increase in proline levels that positively correlate with antioxidant capacity indices, emphasizing their synergistic function in alleviating osmotic and oxidative damage. Similarly, soluble sugars such as glucose, sucrose, and trehalose accumulate in response to salt stress, serving dual functions: osmo-protection through the stabilization of cellular structures and enzymes, and scavenging reactive oxygen species to prevent oxidative damage [43,44]. The reversible water-binding capacity of certain sugars like trehalose provides an additional layer of protection against osmotic imbalance [36]. These osmolytes collectively lower the cellular osmotic potential, facilitating continued water uptake despite the reduced water availability caused by saline conditions. The positive associations observed between osmolyte content and antioxidant activity in our study reinforce the concept that the interplay between osmotic adjustment and antioxidant defense forms an integrated stress mitigation strategy, safeguarding cellular integrity and ensuring physiological functionality during prolonged salt exposure.

Concurrently, salinity induces oxidative stress characterized by excessive reactive oxygen species (ROS) generation, which damages lipids, proteins, and nucleic acids [15,45]. Malondialdehyde (MDA), a reliable marker of lipid peroxidation, showed a strong negative association with agronomic performance, underscoring the detrimental impact of oxidative damage on yield formation under salt stress. In contrast, this decline was counteracted by a robust activation of antioxidant defenses, as evidenced by the accumulation of total phenolics, enhanced radical scavenging activities (DPPH and ABTS), and increased α-tocopherol levels, collectively contributing to cellular protection and yield stability. These antioxidants act as frontline protectants, neutralizing ROS and limiting cellular damage [46]. Partial correlation and redundancy analyses revealed that these biochemical defenses are tightly linked to salinity tolerance and yield preservation. Although individual phenolic acids displayed weaker direct associations with yield traits, their contribution to the overall phenolic pool suggests an indirect yet essential role in stress mitigation. Phenolic compounds constitute a major class of non-enzymatic antioxidants whose effectiveness largely derives from the presence of hydroxyl groups capable of donating electrons to quench free radicals and interrupt oxidative chain reactions [47,48]. Under salinity stress, enhanced accumulation of phenolics reflects an adaptive metabolic reprogramming aimed at reinforcing cellular redox homeostasis [49,50]. Our results corroborate recent findings indicating that stress conditions stimulate phenolic biosynthesis, thereby strengthening antioxidant capacity and protecting cellular components such as membranes, proteins, and nucleic acids from oxidative injury [22,29,51]. The strong positive associations observed between total phenolics and radical scavenging activities (DPPH and ABTS) highlight the functional relevance of these compounds in detoxifying ROS generated under saline conditions.

The activation of antioxidant capacity under stress is closely linked to the regulation of the phenylpropanoid pathway, which governs the synthesis of phenolic acids and flavonoids [49,52]. Salinity stress is known to modulate the activity of key enzymes involved in this pathway, leading to enhanced production of secondary metabolites with protective functions [53]. The variability in phenolic accumulation observed among genotypes in the present study suggests a strong genetic control over these biosynthetic processes, which may partly explain differences in stress tolerance. Genotypes revealing higher antioxidant activity appear better equipped to counteract oxidative pressure, thereby preserving physiological performance and yield-related traits under salinity [54,55].

Importantly, while individual phenolic acids showed limited direct correlations with agronomic traits, their collective contribution to total phenolic content likely underpins a cumulative antioxidant effect. This supports the notion that stress tolerance relies not on single metabolites but on an integrated antioxidant network, where the overall balance and capacity of redox-active compounds determine stress resilience. In this context, the tight coupling between phenolic accumulation, radical scavenging activity, and yield stability observed in our multivariate analyses underscores the central role of antioxidant metabolism in sustaining barley productivity under saline environments. This integrated response facilitates the mitigation of osmotic and oxidative stress effects, ultimately sustaining yield components such as thousand-grain weight (TGW) and main spike seed number (MSGN). Flowering represents a highly sensitive developmental window in cereals, during which spikelet primordia differentiation and survival ultimately determine sink capacity and final yield [56]. At the flowering stage, corresponding to the green anther phase, spikelet primordia undergo rapid cell division and differentiation, processes that are particularly vulnerable to salinity-induced osmotic and ionic constraints [32,57]. Consistent with this physiological sensitivity, flowering date (FD) emerged in our correlation and redundancy analyses as a central trait linking antioxidant capacity with yield-related components. Elevated salinity during this stage intensified oxidative pressure, which can disrupt assimilate allocation to developing spikelets and promote partial spikelet abortion, thereby reducing floret fertility and grain number [57,58]. Our multivariate analyses highlight the pivotal roles of antioxidant compounds and phenolic acids in mediating tolerance mechanisms at this stage. α-Tocopherol, a potent lipophilic antioxidant, emerges as a key protector of photosynthetic membranes and reproductive tissues. By scavenging reactive oxygen species (ROS) and preventing lipid peroxidation, it stabilizes chloroplast membranes under salt-induced oxidative stress, ensuring continued photosynthesis despite stomatal limitations [59]. Its presence in reproductive tissues likely safeguards gametes and developing florets from oxidative injury, crucial for maintaining grain set and yield [59]. Beyond α-tocopherol, several phenolic acids, including ferulic, p-coumaric, syringic, protocatchuic, and quinic acids, play complementary roles in structural and biochemical defenses [22]. Ferulic acid is well-known for its capacity to cross-link with cell wall polysaccharides, enhancing cell wall rigidity and hydrophobicity. This likely limits the apoplastic movement of Na^+^ and Cl^−^ ions into sensitive reproductive cells, aiding ion exclusion and reducing salt toxicity. Additionally, this reinforcement may improve cell wall water retention, mitigating osmotic dehydration during salt stress [60]. *p*-Coumaric acid similarly contributes to strengthening the cell wall matrix, acting as a precursor in lignin biosynthesis. This lignification process can further impede ion penetration and bolster mechanical stability, helping maintain tissue integrity under stress [61]. Syringic acid, with antioxidant properties, likely scavenges free radicals within both cell walls and intracellular compartments, complementing enzymatic antioxidant systems. Its role may extend to signaling pathways modulating stress-responsive gene expression during flowering [62]. Protocatchuic acid is recognized for its radical scavenging activity and metal chelation, potentially reducing oxidative damage by limiting metal-catalyzed ROS generation. It might also participate in modulating stomatal behavior, indirectly influencing transpiration and water use efficiency [22]. Quinic acid, a key intermediate in the shikimate pathway, contributes to phenolic metabolism and synthesis of other bioactive compounds. Its elevated presence may reflect an upregulated defense metabolism, sustaining phenolic pools necessary for antioxidative and structural functions [63]. Together, these phenolic acids orchestrate a multi-layered defense system: structurally fortifying cell walls to restrict ion toxicity and water loss and biochemically scavenging ROS to preserve cellular homeostasis. This is supported by the observed maintenance of relative water content and physiological parameters in salt-stressed plants during flowering.

Importantly, RDA positioned FD in close association with antioxidant-related traits, including total phenolics, radical scavenging activities (DPPH and ABTS), and α-tocopherol, while showing negative relationships with oxidative damage markers such as MDA. This multivariate configuration indicates that genotypes capable of mounting strong antioxidant defenses at flowering are more effective in safeguarding spikelet primordia against oxidative injury, thereby preserving floral organ integrity and reproductive success. In contrast, genotypes with weaker antioxidant responses displayed altered flowering dynamics, increased spikelet abortion, and reduced reproductive performance under salinity stress.

The strong correlations between FD, thousand-grain weight, and main spike seed number further highlight the cascading impact of flowering-stage stress on subsequent grain filling. Salinity-induced perturbations during FD constrain carbon assimilation and translocation during early kernel development, ultimately leading to reduced endosperm deposition and lower seed weight. These results collectively identify FD, together with its associated antioxidant traits, as a critical physiological node governing spikelet survival, grain filling efficiency, and yield stability under salinity.

Genotype-specific correlation patterns revealed contrasting physiological strategies underlying yield performance under stress conditions. In Ardhoui and Kounouz, strong negative correlations between yield components and oxidative stress markers, osmolytes and antioxidant activities indicate that extensive activation of stress-response pathways is associated with yield reduction, reflecting stress-sensitive behavior. Lemsi displayed an intermediate response, with heterogeneous trait–yield relationships and limited yield maintenance despite positive associations between some phenolic compounds and TGW. In contrast, Manel showed a tolerance-associated profile characterized by strong positive relationships between yield components and plant water status, alongside weaker associations with stress-related biochemical traits, suggesting improved yield stability through physiological homeostasis. Rihane showed a genotype-specific pattern in which selected phenolic compounds were positively associated with yield components, although oxidative stress markers remained negatively correlated with yield. Overall, stress tolerance was associated with yield maintenance and reduced dependence on stress-induced metabolic responses rather than enhanced antioxidant activity.

Within this context, the predictive power of a limited subset of physiological and antioxidant traits revealed by partial least squares regression (PLSR) provides quantitative validation of the flowering date (FD)-centered mechanisms identified above. The significant redundancy analysis (RDA) axis highlights that specific explanatory variables strongly influence the variation observed in the response traits, reflecting underlying physiological processes linked to plant stress responses and yield components. The nonsignificance of subsequent axes suggests that the primary drivers of variation were effectively captured by the first axis. The PLSR models offered robust predictive frameworks, with moderate to high coefficients of determination (R^2^ ranging from 0.574 to 0.935) and acceptable RMSEP values, demonstrating good model fit and predictive capacity. Variable Importance in Projection (VIP) analysis further emphasized the key biochemical and physiological traits driving yield variation, consistent with known mechanisms of stress tolerance and plant development. These findings underscore the utility of multivariate approaches in dissecting complex trait relationships in crop performance studies.

The complementary integration of correlation analysis, principal component analysis (PCA), RDA, and PLSR provided a holistic interpretation of barley responses to salinity, whereby RDA highlighted FD and its associated antioxidant traits as salinity-responsive drivers of yield stability, and PLSR confirmed their strong predictive capacity for agronomic performance. This multilevel analytical framework thus reinforces the relevance of flowering-stage antioxidant protection as a key selection target and demonstrates the value of integrating biochemical, physiological, and agronomic traits to dissect complex stress tolerance mechanisms and support breeding for salt-tolerant barley.

## 4. Materials and Methods

### 4.1. Plant Material, Growth Conditions, and Salinity Treatments

Based on their agronomic performance and yield stability under stress conditions, five six-rowed spring barley (*Hordeum vulgare* L.) genotypes, Ardhaoui, Kounouz, Lemsi, Manel, and Rihane, conserved in the Tunisian Gene Bank, were selected for this study. Ardhaoui is a locally adapted barley landrace from southern Tunisia, whereas Kounouz, Lemsi, Manel, and Rihane are registered Tunisian barley varieties selected for comparative evaluation under salinity stress. The experiment was conducted at the Experimental Station of Arid Lands in Medenine, Tunisia (33°61′22″ N, 10°23′47″ E), under controlled greenhouse conditions at the Arid Lands Institute. Temperatures were maintained at 20 ± 2 °C during the day and 16 ± 2 °C at night, with an average relative humidity of approximately 44%. Lighting was provided by yellow lamps under a 16 h light/8 h dark photoperiod.

For each genotype, three independent batches of 96 seeds were sown in 96-well trays. During the first two weeks, all three batches were irrigated with tap water while the seedlings developed up to the two-leaf stage. Starting at this stage, two batches were irrigated with NaCl solutions at 2 g L^−1^, while the third batch continued to receive tap water as the control. Salinity was imposed gradually as detailed in Supplementary Table S1. One week later, at the three-leaf stage, uniform seedlings were transplanted into individual plastic pots (14 cm diameter) filled with a standard growth substrate (Supplementary Table S3). For each genotype × treatment, 10 seedlings were transplanted individually (one plant per pot), yielding 10 biological replicates. Each pot was irrigated with approximately 300–400 mL of solution per session, 2 to 3 times per week, maintaining substrate moisture near field capacity. Irrigation solutions contained NaCl at target concentrations. Pots were individually positioned on greenhouse benches designed to prevent water and ion exchange between experimental units, and irrigation and drainage were managed separately for each pot to avoid lateral sodium migration. Pots were randomized and periodically rotated inside the greenhouse to minimize positional effects.

Physiological and biochemical measurements were performed at the flowering stage on the same plants, while yield-related traits were assessed at maturity. Sampling dates corresponding to the flowering stage of each genotype and treatment are detailed in Supplementary Table S2. Throughout the experiment, standard agronomic practices were applied to avoid confounding effects.

### 4.2. Sampling and Yield-Related Analyses

Physiological and biochemical measurements were performed at the flowering stage (FD), a key developmental phase closely associated with yield formation and highly sensitive to salinity stress. Fully expanded flag leaves were harvested from control and salt-treated plants of each genotype. Samples were immediately frozen in liquid nitrogen and stored at −80 °C until further analyses.

At maturity, yield-related traits, including thousand grain weight, main spike grain number, and main spike grain weight, were recorded to assess the impact of salinity stress on reproductive performance.

### 4.3. Stress-Related Physiological Traits

#### 4.3.1. Relative Water Content (RWC)

Leaf relative water content (RWC) was used as an indicator of plant water status and tissue hydration under salinity stress. Freshly harvested barley leaves (*n* = 10 per treatment) were immediately weighed to determine fresh weight (FW). The leaves were then floated on distilled water for 4 h at room temperature to allow full rehydration, after which the turgid weight (TW) was recorded. Subsequently, the samples were oven-dried at 80 °C for 24 h to obtain dry weight (DW). RWC was calculated using the following equation:

RWC (%) = [(FW − DW)/(TW − DW)] × 100.

#### 4.3.2. Proline Content Determination

Proline accumulation was quantified following a colorimetric assay adapted from established protocols. Briefly, 100 mg of frozen barley leaf tissue were finely ground and homogenized in 1.8 mL of 3% (*w*/*v*) sulfosalicylic acid. The homogenate was centrifuged at 14,000 rpm for 10 min, and 1.5 mL of the resulting supernatant was transferred into screw-cap glass tubes. An equal volume (1.5 mL) of glacial acetic acid and 1.5 mL of freshly prepared ninhydrin reagent were added. The reaction mixture was incubated in a boiling water bath (100 °C) for 1 h. After cooling, 3 mL of toluene were added, and the mixture was vigorously shaken to allow phase separation. The absorbance of the upper organic phase was measured at 520 nm using a Shimadzu UV-1600 spectrophotometer (Shimadzu, Kyoto, Japan). Proline concentration was determined from a standard curve and expressed on a fresh weight basis.

#### 4.3.3. Soluble Sugar Content

Total soluble sugars (glucose and fructose equivalents) were determined using the anthrone method with minor modifications. Fresh barley leaves were homogenized in 2 mL of 80% (*v*/*v*) ethanol and incubated at 75 °C for 40 min. After centrifugation at 10,000× *g* for 5 min at 4 °C, the supernatant was collected, and the extraction was repeated once. The pooled supernatants were evaporated to dryness, and the residue was resuspended in distilled water. For color development, aliquots of the extract were mixed with anthrone reagent (2 g L^−1^ in 75% sulfuric acid), heated at 100 °C for 8 min, rapidly cooled, and absorbance was recorded at 625 nm. Soluble sugar concentrations were calculated using a glucose standard curve and expressed as mg g^−1^ fresh weight.

#### 4.3.4. Lipid Peroxidation Assay (Malondialdehyde Content)

Lipid peroxidation was evaluated by quantifying malondialdehyde (MDA) using the thiobarbituric acid reaction. Leaf tissue (100 mg) was homogenized in 0.1% (*w*/*v*) trichloroacetic acid and centrifuged at 10,000× *g* for 15 min at 4 °C. An aliquot of the supernatant was mixed with thiobarbituric acid solution and incubated at 90 °C for 20 min. The reaction was terminated by rapid cooling, followed by centrifugation. Absorbance was measured at 532 nm and corrected for nonspecific absorbance at 600 nm. MDA concentration was calculated using an extinction coefficient of 155 mM^−1^ cm^−1^ and expressed as nmol g^−1^ fresh weight.

### 4.4. Stress-Related Biochemical Traits

#### 4.4.1. Preparation of Plant Extracts

Phenolic compounds and antioxidant-related metabolites were extracted from barley leaves following a modified maceration protocol adapted from established methods. Briefly, 1 g of finely ground leaf material from each genotype was suspended in 10 mL of methanol and homogenized using an Ultra-Turrax system (IKA®-Werke GmbH & Co. KG, Staufen, Germany). The mixtures were continuously agitated at room temperature for 24 h, followed by centrifugation at 3000 rpm for 30 min. The resulting supernatants were concentrated under reduced pressure using a rotary evaporator and subsequently freeze-dried. Dried extracts were stored at 4 °C in airtight, light-protected vials until further analyses.

#### 4.4.2. Identification and Quantification of Individual Phenolic Compounds

Phenolic profiles were analyzed using liquid chromatography–mass spectrometry (LC–ESI–MS). Lyophilized extracts (20 mg) were re-dissolved in 1 mL of methanol and filtered through a 0.45 µm membrane filter prior to injection. Analyses were performed using a Shimadzu LC–MS 2020 system equipped with an electrospray ionization source operating in negative ion mode. Separation was achieved on a C18 column using a binary gradient of acidified water and methanol. Phenolic compounds were identified by comparing retention times and mass spectra with authenticated standards. Quantification was expressed as µg g^−1^ dry weight (DW).

#### 4.4.3. Total Polyphenol Content (TPC)

Total polyphenol content was determined using the Folin–Ciocalteu colorimetric assay. Absorbance was measured spectrophotometrically, and total phenolics were quantified based on a gallic acid calibration curve. Results were expressed as milligrams of gallic acid equivalents per gram of dry matter (mg GAE g^−1^ DW).

#### 4.4.4. Total Flavonoid Content (TFC)

Total flavonoid content was assessed using the aluminum chloride colorimetric method, with catechin as the reference compound. After color development, absorbance was measured at 510 nm, and flavonoid concentration was expressed as milligrams of catechin equivalents per 100 g of dry matter (mg CE 100 g^−1^ DW).

#### 4.4.5. Condensed Tannin Content

Condensed tannins were quantified using the vanillin–HCl assay. Extracts were reacted with vanillin reagent under acidic conditions, and absorbance was recorded at 500 nm. Catechin was used for calibration, and results were expressed as mg catechin equivalents per 100 g of dry matter.

#### 4.4.6. Antioxidant Capacity Assays

##### DPPH Radical Scavenging Activity

Free radical scavenging capacity was evaluated using the DPPH assay. Extract aliquots were mixed with a DPPH methanolic solution and incubated in the dark. Absorbance was measured at 515 nm, and antioxidant activity was expressed as percentage inhibition relative to the control.

##### ABTS Radical Cation Scavenging Activity

ABTS radical scavenging activity was assessed using a pre-formed ABTS^+^ solution. After reaction with the extracts, absorbance was measured at 414 nm. Antioxidant capacity was expressed as ascorbic acid equivalent antioxidant capacity (AEAC; mg g^−1^ DM).

##### α-Tocopherol Determination

α-Tocopherol content was determined following extraction in phosphate buffer, saponification, and hexane partitioning. After evaporation of the organic phase, the residue was dissolved in ethanol and reacted with 2,2′-dipyridyl reagent. Absorbance was measured at 520 nm, and α-tocopherol concentration was calculated from a standard curve and expressed as µg g^−1^ fresh weight.

### 4.5. Statistical and Multivariate Analyses

All statistical analyses were performed using R software (RStudio environment, version 2025.09.1+401). Data were first subjected to descriptive statistical analysis to evaluate central tendency and variability of biochemical, physiological, and agronomic traits across genotypes and salinity treatments. Prior to inferential analyses, data normality and homogeneity of variances were verified when required. For each trait, a two-way ANOVA was conducted using the model Y = μ + Genotype + Treatment + Genotype × Treatment + ε, with genotype and treatment as fixed factors. Model assumptions were verified graphically, and when necessary, data were transformed prior to analysis. Mean separation was performed using Tukey’s HSD test (*p* < 0.05). Results are presented as mean ± standard deviation for each genotype–treatment combination.

Relationships among biochemical, physiological, antioxidant, and yield-related traits were assessed using Pearson correlation coefficients computed on complete observations. Correlation matrices were visualized using correlograms and network representations, retaining only associations above an absolute threshold of |r| ≥ 0.30. Edge color indicates correlation sign, and edge thickness reflects correlation strength. In addition to pooled analyses, Pearson correlation analyses between physiological, biochemical, antioxidant, and yield-related traits were performed separately for each genotype to identify genotype-specific trait–yield relationships. Genotype-wise correlation matrices are provided as Supplementary Materials. PCA was performed using the prcomp function on standardized variables to explore multivariate trait structure and sample distribution under salinity stress. Biplots were generated for variables and individuals, with samples grouped according to treatment.

RDA was conducted using the vegan package (RStudio environment, version 2025.09.1+401). to relate yield traits (response matrix) to antioxidant and physiological parameters (explanatory matrix). Model significance was evaluated using permutation-based ANOVA (999 permutations) for the global model and individual canonical axes. A partial RDA, conditioning on treatment, was used to assess trait relationships independent of treatment effects. Variables contributing most to ordination patterns were identified based on vector length in RDA space.

PLSR was applied to model relationships between antioxidant/physiological traits (X matrix) and yield components (Y matrix) using the pls package (RStudio environment, version 2025.09.1+401). Models were validated using internal leave-one-out cross-validation, and the optimal number of components was selected based on the minimum RMSEP. Model structure was examined using score and loading plots.

## 5. Conclusions

This study highlights the critical role of coordinated antioxidant defenses, osmotic regulation, and water status maintenance in conferring salinity tolerance and yield stability in barley. By integrating multivariate analyses, we identified key biochemical and physiological traits, such as phenolic compounds, radical scavenging activities, proline accumulation, and relative water content, that serve as robust predictors of performance under salt stress. These findings provide valuable physiological markers and mechanistic insights to guide future breeding strategies aimed at improving barley resilience in saline environments.

## Figures and Tables

**Table 1:** Variation in Individual Phenolic Acids in Barley Genotypes under Salinity Stress as Determined by HPLC–MS (µg g^−1^ dry weight (DW)). Two salinity levels were investigated: moderate salinity (S1, 6 g L^−1^ NaCl) and high salinity (S2, 12 g L^−1^ NaCl). The analyzed compounds include protocatechuic acid, quinic acid, syringic acid, trans-ferulic acid, and p-coumaric acid. Genotypes correspond to G1 (Ardhaoui), G2 (Kounouz), G3 (Lemsi), G4 (Manel), and G5 (Rihane).

Treatment	Genotype	Protocatechuic	Quinic	Syringic	Trans-Ferulic	*p*-Coumaric
C	G1	86.78 ± 1.52 a	14,450.63 ± 619.58 g	37.08 ± 0.3 bc	796.79 ± 7.38 d	231.66 ± 3.78 a
S1	G1	45.69 ± 0.95 fgh	42,857.62 ± 1678.12 b	37.49 ± 0.83 bc	599.03 ± 2.87 ef	99.26 ± 2.42 g
S2	G1	38.25 ± 0.62 hi	28,442.78 ± 572.54 de	26.42 ± 1.6 d	1142.56 ± 66.99 a	90.86 ± 4 g
C	G2	52.52 ± 1.41 ef	51,325.47 ± 383.04 a	25.44 ± 0.61 d	616.52 ± 7.53 ef	110.03 ± 3.37 efg
S1	G2	42.29 ± 0.53 gh	32,019.4 ± 990.2 cd	25.28 ± 1.16 d	561.87 ± 18.63 f	97.2 ± 1.41 g
S2	G2	61.18 ± 2.9 cd	42,276.6 ± 2131.57 b	41.45 ± 0.75 ab	958.95 ± 23.16 b	130.53 ± 0.61 de
C	G3	46.04 ± 0.99 fg	22,993.5 ± 1374.86 ef	22.64 ± 0.68 de	875.01 ± 3.51 bcd	105.09 ± 2.46 fg
S1	G3	43.8 ± 0.72 gh	38,098.35 ± 2993.9 bc	10.55 ± 0.42 g	919.84 ± 33.08 bc	221.27 ± 12.09 ab
S2	G3	75.46 ± 2.49 b	22,505.69 ± 557.26 ef	43.94 ± 0.85 a	870.08 ± 13.3 bcd	122.17 ± 1.02 def
C	G4	64.41 ± 0.62 c	20,481.49 ± 476.08 fg	32.95 ± 0.4 c	541.33 ± 1.02 f	102.52 ± 1.2 fg
S1	G4	49 ± 2.26 efg	31,993.46 ± 746.79 cd	13.45 ± 0.67 fg	836.63 ± 4.79 cd	201.65 ± 4.92 b
S2	G4	56.14 ± 1.38 de	19,171.39 ± 1420.81 fg	27.03 ± 0.88 d	682.13 ± 5.49 e	156.64 ± 5.74 c
C	G5	32.76 ± 0.45 i	31,228.43 ± 564.86 d	35.4 ± 0.89 c	528.76 ± 5.91 f	99.73 ± 1.44 fg
S1	G5	46.43 ± 1.23 fg	32,407.95 ± 891.47 cd	17.84 ± 1.99 ef	609.79 ± 1.15 ef	139.45 ± 3.64 cd
S2	G5	38.47 ± 0.42 hi	40,521.68 ± 428.51 b	15.05 ± 0.78 fg	598.04 ± 3.02 ef	105.76 ± 1.99 fg

Values are expressed as mean ± standard error (SE) of ten biological replicates (*n* = 10). Data were analyzed using two-way ANOVA. When significant effects were detected, mean separation was performed using Tukey’s HSD post hoc test at *p* < 0.05. Different lowercase letters within the same column indicate statistically significant differences among genotype × treatment combinations.

## Data Availability

The datasets used and analyzed during the current study available from the corresponding author on reasonable request.

## Supplementary Materials

The following supporting information can be downloaded at: https://www.mdpi.com/article/10.3390/ijms27052454/s1.
