# Supplementary material for: Coordinated Antioxidant and Physiological Responses at Flowering Promote Yield Stability in Salinity-Stressed Barley Genotypes
Source: Int J Mol Sci. 2026 Mar 7;27(5):2454. doi: 10.3390/ijms27052454 (PMC12986377; doi:10.3390/ijms27052454)
Supplement: Supplementary file 1 [file ijms-27-02454-s001.zip › ijms-4106098-supplementary.pdf]

1. Figures

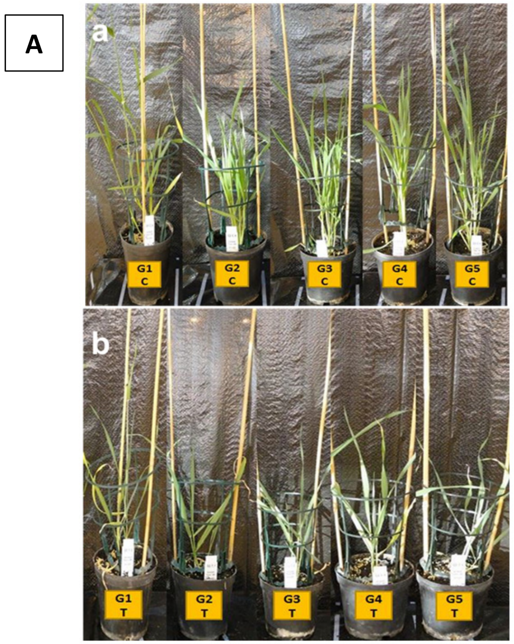

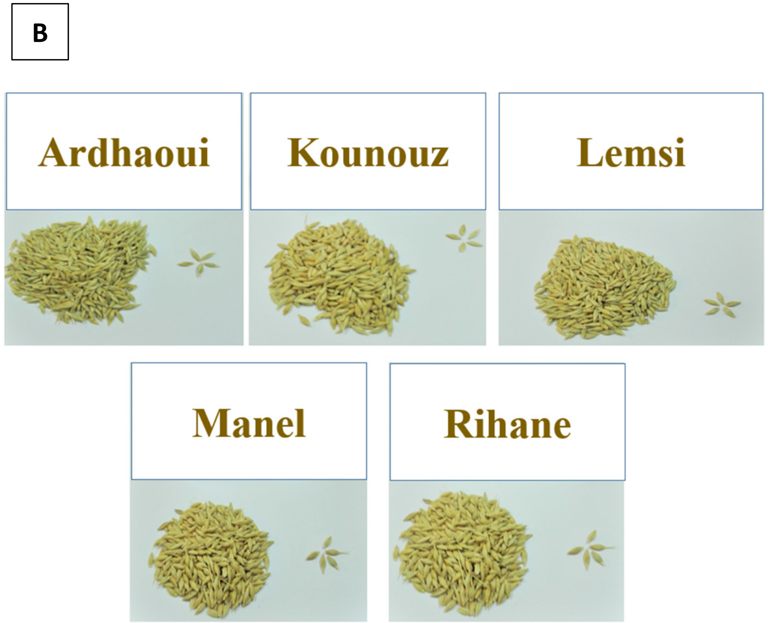

Figure S1. (A)Representative photographs of barley genotypes under control (a) and salinity stress conditions (b), illustrating contrasting growth responses between tolerant and sensitive genotypes. (B) Studied barley grains

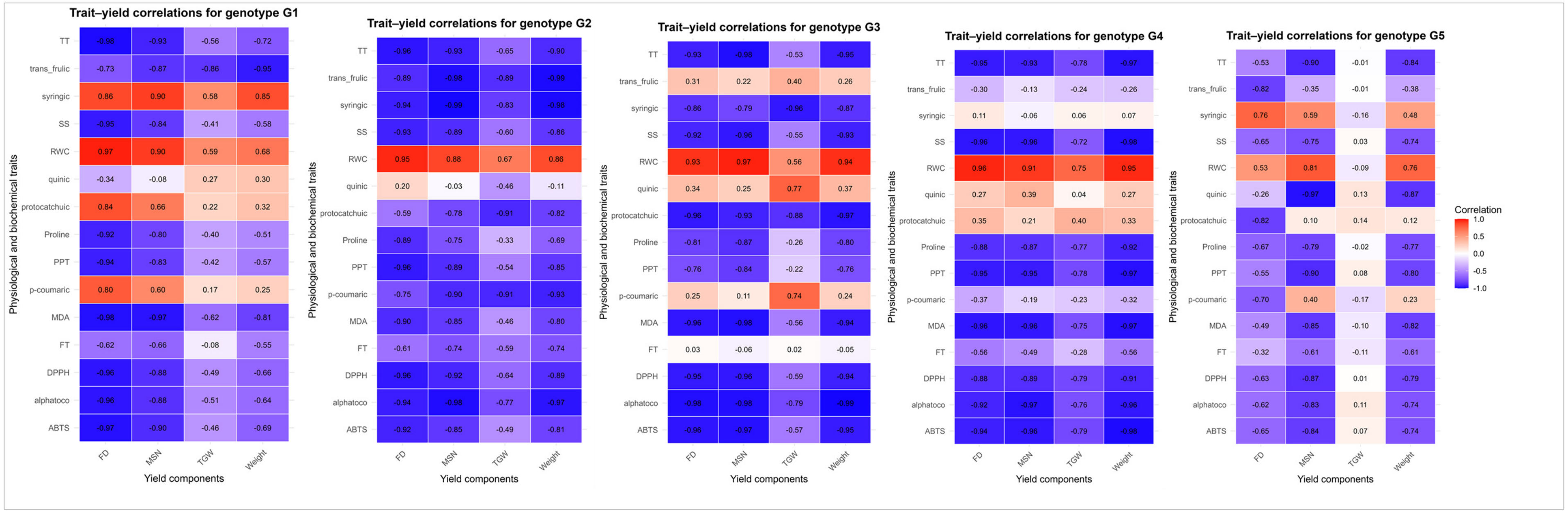

Figure S2. Genotype-specific correlations between physiological, biochemical traits and yield components under stress conditions. Heatmaps of Pearson correlation coefficients between physiological, biochemical traits and yield components calculated separately for each genotype (G1–G5) under stress conditions. Yield components include Flowering Date (FD), main spike grain number (MSGN), thousand-grain weight (TGW) and main spike grain weight (MSGW). Physiological and biochemical traits include relative water content (RWC), soluble sugars (SS), proline, malondialdehyde (MDA), antioxidant activities (DPPH, ABTS and α-tocopherol), and phenolic compounds (quinic, protocatechuic, syringic, p-coumaric and trans-ferulic acids), as well as total phenolic content (PPT) and total flavonoids (FT). Correlation coefficients are indicated by the colour scale, with red and blue colours representing positive and negative correlations, respectively, and color intensity reflecting correlation strength (−1 to +1). The analysis reveals genotype-dependent trait–yield relationships, highlighting contrasting correlation patterns between stress-related traits and yield components among genotypes.

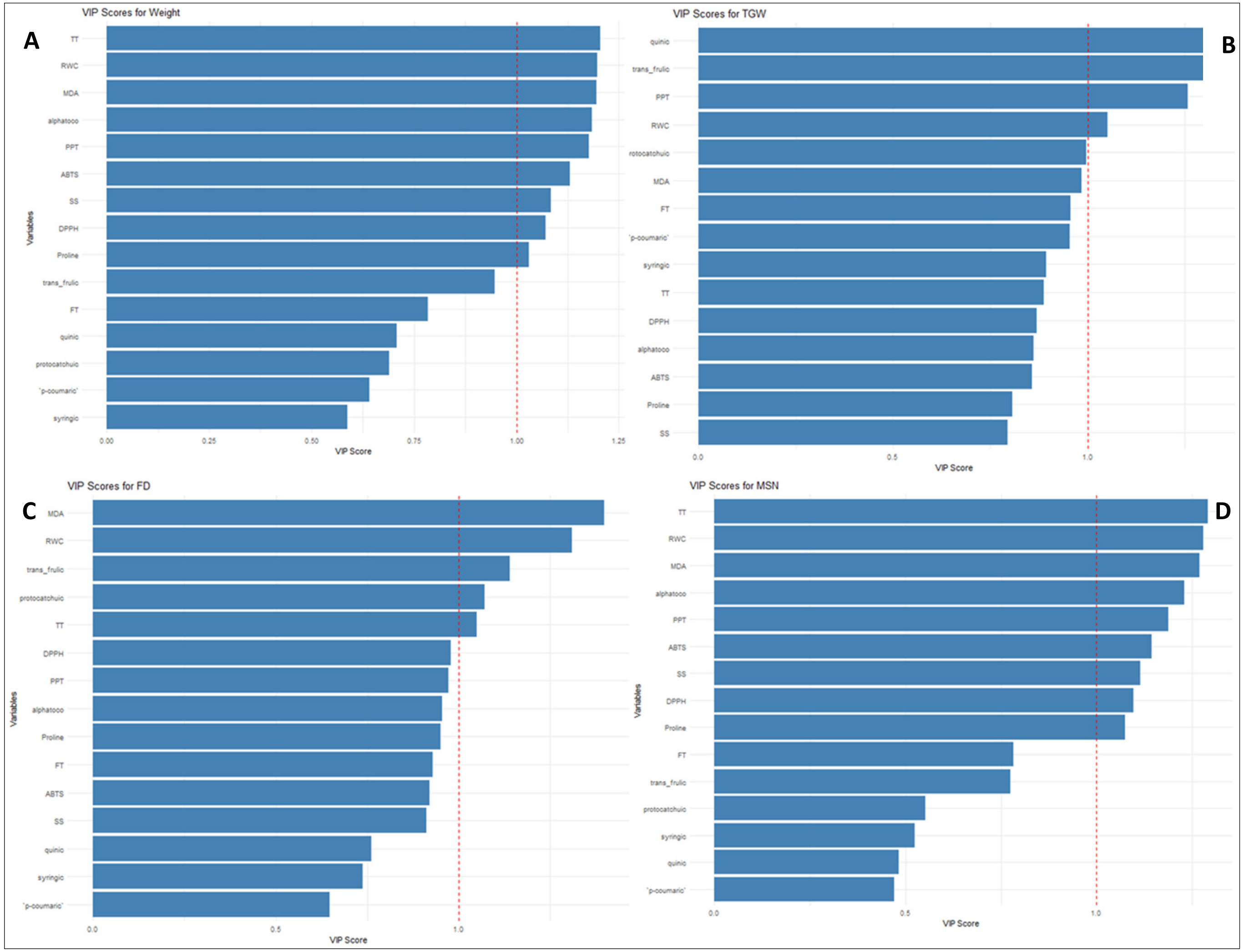

Figure S3. Variable Importance in Projection (VIP) Scores for Key Traits in Barley Genotypes The VIP scores indicate the contribution of each biochemical and physiological variable to the variation in four important traits: (A) MSGW, (B) Thousand Grain Weight (TGW), (C) Flowering Date (FD), and (D) Main Spike Grain Number (MSGN). Variables with VIP scores above the red dashed line (score = 1) are considered significant contributors to the respective trait. These scores help identify the most influential parameters associated with each trait under the studied conditions.

2. Tables

Table 1. Weekly Salt Concentrations Used for Gradual Salt Stress Imposition on Barley genotypes

week	Moderate salinity (6g/l NaCl)	Severe salinity (12g/l NaCl)
1	0 (tap water)	0 (tap water)
2	0 (tap water)	0 (tap water)
3	2	2
4	4	4
6	6	6
7	6	8
8	6	10
9	6	12
10	6	12

Table 2. Flowering and Sampling Dates for Barley Genotypes Under Salinity Treatments

Treatment	Genotype	Rep	FD
C	G1	R1	58
C	G1	R2	57
C	G1	R3	57
C	G2	R1	56
C	G2	R2	57
C	G2	R3	53
C	G3	R1	48

C	G3	R2	52
C	G3	R3	51
C	G4	R1	51
C	G4	R2	53
C	G4	R3	54
C	G5	R1	59
C	G5	R2	58
C	G5	R3	64
S1	G1	R1	52
S1	G1	R2	53
S1	G1	R3	54
S1	G2	R1	54
S1	G2	R2	51
S1	G2	R3	51
S1	G3	R1	49
S1	G3	R2	48
S1	G3	R3	46
S1	G4	R1	51
S1	G4	R2	50
S1	G4	R3	49
S1	G5	R1	52

S1	G5	R2	51
S1	G5	R3	54
S2	G1	R1	45
S2	G1	R2	47
S2	G1	R3	44
S2	G2	R1	43
S2	G2	R2	45
S2	G2	R3	44
S2	G3	R1	33
S2	G3	R2	32
S2	G3	R3	34
S2	G4	R1	44
S2	G4	R2	46
S2	G4	R3	45
S2	G5	R1	54
S2	G5	R2	56
S2	G5	R3	54

Table. 3 Soil physical and chemical properties before planting

**Attribute**	**Soil**	**Unit**
**pH**	7.9	-

**Electrical Conductivity (EC)**	7.12	dS cm^{-1}
**Organic Matter (MO)**	0.69	%
**Nitrogen (N)**	1.14	%
**Sodium (Na)**	13.94	Mg kg^{-1}
**Potassium (K)**	61.42	Mg kg^{-1}
**Phosphorus (MG/KG)**	52.04	ppm
**Calcium (Ca)**	839	Mg kg^{-1}
